# Tracking of Medicine Use and Self-Medication From Infancy to Adolescence: 1993 Pelotas (Brazil) Birth Cohort Study

**DOI:** 10.1016/j.jadohealth.2012.06.027

**Published:** 2012-12

**Authors:** Andréa Dâmaso Bertoldi, Marysabel Pinto Telis Silveira, Ana M.B. Menezes, Maria Cecília Formoso Assunção, Helen Gonçalves, Pedro Curi Hallal

**Affiliations:** a Postgraduate Program in Epidemiology, Federal University of Pelotas, Pelotas, Brazil; b Pharmacy Course, Federal University of Pampa, Campus Uruguaiana, Brazil

**Keywords:** Drug use, Self-medication, Cohort studies, Adolescent, Child

## Abstract

**Purpose:**

To track the use of medicine and self-medication from infancy to adolescence.

**Methods:**

All newborns in the city of Pelotas, Brazil, were monitored and enrolled in the 1993 Pelotas (Brazil) Birth Cohort Study. Subsamples of the cohort were visited at 1, 3, and 6 months and at 1 and 4 years of age. At 11 and 15 years of age, all cohort members were sought. In each of these visits, information on medicine use in the 15 days before the interview was collected. Two outcomes were analyzed: prevalence of medicine use and prevalence of self-medication.

**Results:**

Prevalence of medicine use in the first year of life ranged from 52.0% to 68.6%. From 4 to 15 years of age, the prevalence decreased, ranging from 30.9% to 37.2%. At age 15, girls presented a 33% higher prevalence of medicine use than boys. The proportion of self-medication ranged from 12.4% to 29.0% and was higher in girls aged 11 and 15 years than boys of the same age. In all follow-up periods, use of medicines was significantly higher among children who used medicines in the earlier period. Prevalence of self-medication was higher among those who were self-medicated in the previous periods.

**Conclusions:**

Tracking studies are important to public health because they can predict future behavior by analyzing past behavior, thus helping in designing preventive actions. In this study, previous use of medicines predicts future use, thus creating an opportunity to plan actions that promote the rational use of medicines.

**Implications and Contribution**

In all waves of data collection of this Brazilian study, which followed up >4,000 individuals from birth to adolescence, use of medicines and self-medication tracked strongly from one period to the next one, thus suggesting that preventive strategies should start as early as possible.

Rational use of medicines is of concern, independent of age, and becomes even more important in younger ages because there are significant biological differences between children and adults regarding pharmacokinetics and pharmacodynamics of medicines [1].

The international literature shows that medicine use is highly prevalent among children [2], especially in the first months of life [3], and adolescents [4,5]. The same can be observed in Brazil. According to Moraes et al [6], in the city of Maringá, Brazil, 56% of the adolescents aged 14–18 years had used medicines in the previous 15 days; 53% used these medicines by self-medication, and the majority of these adolescents did not know the names of their medicines. Studies carried out in the city of Pelotas, Brazil, showed a high prevalence of medicine use among children and adolescents; 64% in children aged 1 year [7], 56% in those aged 3–4 years [8], and 31% in those aged 11 years [9]. Consistently, the literature indicates that the prevalence is higher among girls than boys [5,6,10,11].

Worldwide, self-medication is an important public health problem among children and adolescents [3,12]. The prevalence of self-medication among children aged 0–17 years was studied in Germany [13]. It was found that 25.2% of participants had self-medicated in the previous week, corresponding to 38.5% of total medicine use, including all pharmacological classes. Holstein et al [14] described the self-reported medicine use for common health complaints among those aged 11–15 years in Denmark between 1988 and 1998, finding a high proportion of medicine use, and concluded that the proportion of users increased during the 10 years studied. Studies conducted in Brazil found that approximately one-half of medication use in adolescents is by self-medication [11,12,14]. Also, it was observed that self-medication increased according to age from 11% at 3 months to 34% at 2 years [7]. Among those aged 7–18 years, the risk of self-medication was twofold higher compared with children aged <7 years (odds ratio [OR] = 2.81; 95% confidence interval [CI]: 2.09–3.77) [12].

Analyses targeted to observe the likelihood of medicine use in certain period of life in relation to medicine use in previous periods (tracking of medicine use) were not found in the literature. Tracking is defined as the stability of a variable over a period [15,16]. Malina [17] defined the tracking effect as “individual trend in maintaining oneself in a determined position or group after a period of time.” There are several studies in the literature that track other variables, such as food intake [18–20] and physical activity practice along one's life [21]. In the area of pharmacoepidemiology, however, most cohort studies used cross-sectional approaches to evaluate medicine use [2,3,9,14], and just a few used repeated cross-sectional designs to evaluate the use of specific groups of medicines over time [22–25]. The aim of this study was to track the use of medicine and self-medication practices from infancy to adolescence in a Southern Brazilian city.

## Methods

All hospital births in the city of Pelotas, Brazil, in 1993 were monitored. Information was collected using a questionnaire administered by trained interviewers to mothers. In total, 5,265 mothers gave birth in that calendar year in the city, of which 5,249 agreed to take part in the cohort study. Newborns were weighed and measured. Subsamples of the cohort were visited at 1, 3, and 6 months and at 1 and 4 years of age. In each of these visits, information on medicine use by the infant/child in the 15 days before the interview was collected from mothers. At 11 and 15 years of age, all cohort members were searched for follow-up visits; again, data regarding medicine use in the 15 days before the interview were collected from mothers. For reported medicine use, mothers were asked for the name of the medicine, the reason for its use, and whether it had been prescribed by a physician. The sample size of each visit is presented in Table 1. The methodological details of the 1993 Birth Cohort Study are described elsewhere [26].

For this study, we analyzed two outcomes in each of the follow-up visits: (a) prevalence of medicine use and (b) prevalence of self-medication. Self-medication refers to the use of any medicine without a medical prescription. In terms of confounders, we adjusted our analyses for sex, maternal schooling, and family income. For analyses using adolescent data, confounders were maternal schooling and family's assets index collected in adolescence.

We carried out descriptive, crude, and multivariate analyses using Stata 10.0 (Stata Corp., College Station, TX). Analyses using variables collected at 1 and 4 years of age were weighed owing to the oversampling of low-birth-weight children. The descriptive analysis included calculating absolute numbers and percentages for all outcomes, for the whole sample, and separately for boys and girls. As the main objective was to show tracking of medicine use and self-medication, we also presented the prevalence of the outcomes according to the same variable in the previous visit. In these cases, the medicine use at 11 years of age, for example, is both an outcome (the main exposure is medicine use at 4 years of age) and an exposure (the outcome is medicine use at 15 years of age).

In the crude analysis, the prevalence of each outcome was regressed using information on medicine use (or self-medication) in the previous visit as the main exposure. These analyses were carried out using logistic regression model, and ORs and 95% CIs are provided. For the adjusted analyses, the following variables were added to the model: sex, maternal schooling, and family income or assets index. Statistical significance was tested using the χ^2^ test and was set at 5% (all tests were two-tailed).

The study was approved by the Research Ethics Committee of the School of Medicine of the Federal University of Pelotas. Parents or guardians provided written informed consent, authorizing the participation of their child in the study.

## Results

Table 1 shows the prevalence of medicine use in children from the 1993 Birth Cohort Study. In the first year of life, the prevalence was higher (ranging from 52.0% to 68.6%) than from 4 to 15 years of age, in which the prevalence decreased (range: 30.9%–37.2%). Up to age 11, differences in medicine use by sex were not found. At age 15, girls presented a 33% higher prevalence in the use of medicine than boys.

The prevalence of self-medication presented a U-shaped relationship with age. Prevalence values were higher in early life (up to 3 months of age) and at mid-adolescence (15 of age) as compared with late infancy (6 and 12 months of age), mid childhood (4 of age), and early adolescence (11 years of age). Self-medication tended to be higher among girls than boys in most visits, although differences were only statistically significant in adolescence (11- and 15-year follow-ups).

Table 2 shows the prevalence of medicine use according to the frequency of medicine use in the previous follow-up visit. In all studied periods, the prevalence of medicine use was higher among children who used medicines in the earlier period. Considering medicine use in children aged 1 month, 74.6% of them also used medicines at 3 months, whereas 57.5% of those who did not use medicines at the first month of life, used them at 3 months. The same interpretation is applied to the other periods. Crude and adjusted ORs are also shown in Table 2, indicating the increased chance of medicine use in the current study in relation to the previous study. All *p* values were significant, indicating that previous use of medicine predicts use in the future. In the adjusted model, children who used medicines in the first month of life presented a 2.11 times higher likelihood of using medicines at 3 months of age than those who did not use at 1 month of age (OR: 2.11; 95% CI: 1.49–2.99).

Similarly, Table 3 shows the same analyses using self-medication as the outcome. Prevalence of self-medication was higher among those who were self-medicated in the previous periods than those who did not self-medicate. However, in two follow-up visits (1 and 11 years of age), the difference was not statistically significant.

## Discussion

The purpose of evaluating tracking of medicine use from childhood to adolescence is to identify the increased risk of overmedication and to carry out preventive action aimed at increasing the rational use of medicines during this developmental period in life. Considering that adolescence is a developmental stage with special concerns related to behaviors and needs regarding health care, it is important to understand medicine use in this period. Longitudinal analyses carried out using data from birth cohort studies are the ideal source of information on this issue.

In the present study, those who used medicines in the previous period were consistently more likely to use medicines in the following visits, regardless of age, income, and maternal education. The proportion of medicine use among those who had previously used medicines was higher than among those who did not use medicines before, thus one of the most important contributions of this article was to demonstrate that there is a tracking of medicine use from childhood to adolescence. A tracking effect was also observed in the practice of self-medication; those who self-medicated in the past were more likely to continue to do so.

Cohort studies conducted with children and adolescents in the northern region of the Netherlands in 1998 [27], in Denmark between 1988 and 1998 [14], and in the city of Pelotas, Brazil [9], assessed the prevalence of medicine use at different ages. However, these studies used cross-sectional analyses, and therefore did not track the use of medicine over time.

The prevalence of medicine use was higher in the first year of life and decreased with age until the beginning of adolescence, where a small increase was observed, as found in previous international [27] and national studies [28]. In a cohort study performed in the northern region of the Netherlands, Schirm et al [27] reported that the prevalence of medicine use in children aged 1 year was 87%; this prevalence decreased to 51% until 12 years of age, remained stable at around 50% from 6 to 12 years of age, and increased to 64% at 16 years of age.

Bertoldi et al [28] conducted a study in Southern Brazil that included individuals between 0 and 19 years of age living in an area covered by the Family Health Program (Programa de Saúde da Família, PSF). Results of this study found the prevalence of medicine use to be higher in those aged 0–4 years (50.6%), to decrease in children aged 5–9 years (35.2%), and to increase again in those aged 10–19 years (38.7%).

The present study is consistent with the national [6,11] and international [4,5,10] literature regarding the increased use of medicine during adolescence, in which there is evidence of differences by sex in adolescents, with girls having a higher prevalence of medicines use than boys [4–6,10,11].

The prevalence of medicines used at 15 years of age was higher than the prevalence at 11 years of age; these prevalence rates are lower than those found in other studies (40%–56%) with teenagers [4–6,10,11]. The trend of increasing use with age during adolescence is similar to findings from other studies. However, to make comparisons between studies, considerations must be given to the variability in the recall period and the ages of the participants in each study.

Holstein et al [5] conducted research with students aged 11–13 years in six European countries and asked about medicine use for headaches in the past month. The results indicate that 42.5% of the sample had used medicines for headaches during the past month, and this prevalence increased with age and in girls.

Gobina et al [10] studied adolescents aged 11–15 years and medicines used by them for headaches, stomachaches, inability to sleep, and nervousness for a period of 1 month in 19 countries in Europe and in the United States. On average, the most prevalent use of medicine was for headaches (40.9%), followed by stomachaches (23.8%). This study also reported increased medicine use according to age and higher prevalence in girls, for all medicines examined.

De Moraes et al [6] evaluated the prevalence of medicine use in the past 15 days among high school students (14–18 years old) living in an urban area in Southern Brazil in 2007. This study found 55.8% of adolescents took medicine, and the rate was also higher in girls (girls = 64.3%, boys = 45.7%, *p* < .001). Age presented a positive and significant linear trend (*p* = .0001), with the prevalence rate increasing from 50% in those aged 14 years to 57.6% in those aged 16 years.

Furthermore, a study conducted with a representative sample of schoolchildren aged 14–16 years, in Porto Alegre, Southern Brazil, found the consumption of medicine in the past 7 days was 49.5%, with a significantly higher proportion of girls using medicine than boys (57.6% vs. 38.8%, *p* = .001). The same type of upward trend according to age was also observed in this study [11].

As for self-medication, the data confirm that this is a fairly common practice among children and adolescents. The prevalence of self-medication was higher in the first 3 months of life, decreasing to values around 15% until 11 years of age, and at 15 years of age, there was an increase, driven primarily by an increase in self-medication in girls. The difference between sexes in self-medication was evident and has been demonstrated by other authors [4,13,29]. In a study by Hansen et al [4], at 11 and 15 years of age, a statistical significant difference was shown among girls and boys in self-medication. Yong Du and Hildtraud Knopf [13] studied self-medication during the previous week among children and adolescents from 0 to 17 years of age in Germany. The authors found that 25.2% of participants had used self-medication. As in the present study, overall, girls showed a significantly higher level of self-medication than boys, and self-medication increased from 14 to 17 years of age.

The increased use of medicines and self-medication by girls at 15 and 11 years of age, respectively, may be explained by menarche, a period in which girls use analgesics and contraceptives. Hansen et al [4] showed that for girls aged 11–15 years, use of medicines for headaches and stomachaches occurred more often than among boys [4]. Dysmenorrhea is associated with younger age [30], and dysmenorrheal women use numerous drugs by self-medication for pain but infrequently accessed formal medical care [31]. Another explanation is that women typically present greater readiness to acknowledge and express their health needs as compared with men, thus also presenting higher use of medical care [32]. The study by Klein et al reinforces this idea by showing that the proportion of girls who reported using a physician's office was significantly higher than boys [33]. According to Marcell et al, in younger adolescents (11–15 years old), there are no differences between the sexes regarding health care visits; however, in older male subjects, these visits are significantly reduced in comparison with female subjects [32].

In a study by Abahussain et al [34], performed in Kuwait, the prevalence of self-medication among adolescents aged 14–19 years was high (92%), increasing with age from 87% among those aged 14 years to 95% among those aged 18 years; 65% of medicines used were for pain relief. In Brazil, studies show that the prevalence of self-medication in adolescents is approximately 30% [12,35]. In a study by Silva et al [35], conducted in Fortaleza, Ceará, Brazil, with adolescents aged 13–18 years, 34% reported ever having used medicines because of media advertisements.

According to Silva et al [35], teenagers are in a phase of life in which they are formulating views on various social factors, including the use of medicine and other substances. The authors point out the widespread practice of self-medication in adolescence, particularly among girls, envisioning possible permanent medicine users.

Usually in childhood, and often in adolescence, the person who provides the medicine to the child is either the mother or guardian responsible for them. In a study conducted among adolescents [11], one predictor of high medication use in children and adolescents was maternal education. Lower maternal educational was associated with greater likelihood of self-medication. Bertoldi et al [9] found that medicine use was directly associated with economic status and maternal schooling and reported 28% higher levels of self-medication in groups with more household assets and higher maternal schooling [9]. The same study reported a positive association between maternal use of hypnotic drugs and sedatives and adolescent medicine use.

One of the limitations of tracking studies is how to conduct the tracking analysis. Tracking is often estimated by correlation coefficients between two continuous variables collected at different time points or by the proportion of subjects consistently classified in a certain “risk” group at a follow-up measurement. Also, ORs are used to estimate the magnitude of tracking [15]. In addition to this broader limitation, there are specific limitations to the self-report of medicine use and self-medication owing to the likelihood of recall bias. Another problem is the possibility that some mothers were unaware of the use of medicines by the adolescents, particularly contraceptives, as well as medicines used in the treatment of sexually transmitted diseases, for example.

Tracking studies are important from an epidemiological perspective because future behavior may be “predicted” by analyzing the past, thus aiding in the design of preventive actions. In our study, we showed that both medicine use and self-medication track over time. In addition to the tracking effect, the prevalence of medicine use and self-medication was high. Educational programs about safe and rational use of medicines at school level targeted to students and their relatives could be a strategy to prevent abusive use of medicines and irresponsible self-medication. Future studies will benefit from analyzing tracking of use of specific pharmacological groups.

## Figures and Tables

**Table 1:** Medicine use and self-medication among boys and girls 15 days before the interview in infancy, childhood, and adolescence—1993 Pelotas (Brazil) Birth Cohort Study

Follow-up visit	N^a^	Medicine use				Self-medication			
		All (%)	Boys (%)	Girls (%)	*p*	All (%)	Boys (%)	Girls (%)	*p*
1 month	649	64.7	67.4	61.9	.146	29.0	30.5	27.3	.364
3 months	644	68.6	69.9	67.3	.482	25.9	24.4	27.6	.359
6 months	1,413	56.6	54.6	58.4	.147	15.1	13.9	16.9	.044
1 year	1,362	52.0	53.5	50.6	.300	17.0	15.8	18.1	.235
4 years	1,272	35.0	37.4	32.7	.079	15.7	15.4	16.0	.787
11 years	4,426	30.9	30.2	31.6	.326	12.4	10.3	14.5	<.001
15 years	4,341	37.2	31.9	42.3	<.001	20.2	17.3	22.9	<.001

a Only the 11- and 15-year follow-up studies included the entire sample.

**Table 2:** Tracking of medicine use from 1 month to 15 years of age—1993 Pelotas (Brazil) Birth Cohort Study

Follow-up visit	Medicine use in the previous follow-up visit		Unadjusted^a^		Adjusted^b^	
	No (%)	Yes (%)	OR (95% CI)	*p*	OR (95% CI)	*p*
3 months^c^	57.5	74.6	2.17 (1.54–3.07)	<.001	2.11 (1.49–2.99)	<.001
6 months	49.5	62.5	1.70 (1.21–2.39)	.002	1.80 (1.28–2.55)	.001
1 year	44.4	57.8	1.72 (1.38–2.14)	<.001	1.73 (1.39–2.16)	<.001
4 years	30.1	39.5	1.52 (1.20–1.92)	<.001	1.45 (1.15–1.84)	.002
11 years	29.1	37.4	1.45 (1.13–1.88)	.004	1.39 (1.06–1.84)	.017
15 years	32.4	47.8	1.91 (1.67–2.18)	<.001	1.92 (1.68–2.22)	<.001

CI = confidence interval; OR = odds ratio.

a Unadjusted: In these analyses, medicine use in each study was compared with medicine use in the previous period. For example, at 3 months, children who used medicines at the first month of life had a chance to use medicine 2.17-fold higher than children who did not use medicine at the first month.

b Adjusted: Analyses adjusted for sex, maternal schooling, and family income at the time of the birth to studies from 3 months to 4 years. For 11- and 15-year follow-up visits, analyses were adjusted for sex (perinatal data), maternal education at 15 years and asset index at 11 and 15 years.

c The 3-month study was compared with the 1-month study.

**Table 3:** Tracking of self-medication from 1 month to 15 years of age—1993 Pelotas (Brazil) Birth Cohort Study

Follow-up visit	Self-medication in the previous follow-up visit		Unadjusted^a^		Adjusted^b^	
	No (%)	Yes (%)	OR (95% CI)	*p*	OR (95% CI)	*p*
3 months^c^	20.5	39.3	2.50 (1.75–3.63)	<.001	2.34 (1.60–3.42)	<.001
6 months	13.1	29.8	2.82 (1.83–4.34)	<.001	3.02 (1.94–4.72)	<.001
1 year	16.4	21.3	1.38 (.95–2.01)	.086	1.40 (.97–2.04)	.075
4 years	14.7	21.0	1.54 (1.06–2.23)	.024	1.50 (1.03–2.19)	.035
11 years	13.0	17.7	1.44 (.95–2.19)	.088	1.40 (.90–2.19)	.140
15 years	19.1	28.2	1.67 (1.35–2.05)	<.001	1.62 (1.31–2.02)	<.001

a Unadjusted: In these analyses, self-medication in each study was compared with self-medication in the previous period.

b Adjusted: Analyses adjusted for sex, maternal education, and family income at the time of the birth to studies from 3 months to 4 years. For 11- and 15-year follow-up visits, analyses were adjusted for sex (perinatal data), maternal education at 15 years and asset index at 11 and 15 years.

c The 3-month study was compared with the 1-month study.
